# Angiogenesis precedes myogenesis during regeneration following biopsy injury of skeletal muscle

**DOI:** 10.1186/s13395-023-00313-3

**Published:** 2023-02-14

**Authors:** Nicole L. Jacobsen, Aaron B. Morton, Steven S. Segal

**Affiliations:** 1 Department of Medical Pharmacology and Physiology, University of Missouri, Columbia, MO USA; 2 Dalton Cardiovascular Research Center, Columbia, MO USA; 3 Department of Biomedical Sciences, University of Missouri, Columbia, MO USA; 4 Department of Biomedical, Biological, and Chemical Engineering, University of Missouri, Columbia, MO USA

**Keywords:** Skeletal muscle, Biopsy injury, Angiogenesis, Myogenesis

## Abstract

**Background:**

Acute injury to skeletal muscle damages myofibers and fragment capillaries, impairing contractile function and local perfusion. Myofibers and microvessels regenerate from satellite cells and from surviving microvessel fragments, respectively, to restore intact muscle. Established models of injury have used myotoxins and physical trauma to demonstrate the concurrence of myogenesis and angiogenesis during regeneration. In these models, efferocytosis removes cellular debris while basal laminae persist to provide guidance during myofiber and microvessel regeneration. It is unknown whether the spatiotemporal coupling between myofiber and microvascular regeneration persists when muscle tissue is completely removed and local guidance cues are lost.

**Methods:**

To test whether complete removal of skeletal muscle tissue affects the spatiotemporal relationship between myogenesis and angiogenesis during regeneration, subthreshold volumetric muscle loss was created with a biopsy punch (diameter, 2 mm) through the center of the gluteus maximus (GM) in adult mice. Regeneration into the void was evaluated through 21 days post-injury (dpi). Microvascular perfusion was evaluated in vivo by injecting fluorescent dextran into the circulation during intravital imaging. Confocal imaging and histological analyses of whole-mount GM preparations and tissue cross-sections assessed the growth of microvessels and myofibers into the wound.

**Results:**

A provisional matrix filled with PDGFRα^+^ and CD45^+^ cells spanned the wound within 1 dpi. Regenerating microvessels advanced from the edges of the wound into the matrix by 7 dpi. Nascent microvascular networks formed by 10 dpi with blood-perfused networks spanning the wound by 14 dpi. In striking contrast, the wound remained devoid of myofibers at 7 and 10 dpi. Myogenesis into the wound was apparent by 14 dpi and traversed the wound by 21 dpi. Regenerated myofibers and microvessels were disorganized compared to the uninjured muscle.

**Conclusions:**

Following punch biopsy of adult skeletal muscle, regenerating microvessels span the wound and become perfused with blood prior to myofiber regeneration. The loss of residual guidance cues with complete tissue removal disrupts the spatiotemporal correspondence between microvascular and myofiber regeneration. We conclude that angiogenesis precedes myogenesis during regeneration following subthreshold volumetric muscle loss.

**Supplementary Information:**

The online version contains supplementary material available at 10.1186/s13395-023-00313-3.

## Background

Injury to skeletal muscle damages myofibers, triggers an inflammatory response, and fragments capillary networks [1–3]. While the events and time course of the inflammatory cascade [3] and the regeneration of myofibers from muscle stem cells [satellite cells (SCs)] following injury are well defined [1, 4–6], less is known about the regeneration of a functional microcirculation. Following exposure to myotoxins or physical trauma, capillary blood flow is abolished within 1 day post-injury (dpi); angiogenesis begins 2–3 dpi and perfused capillary networks are restored within 5 dpi [1, 7]. Although initially disorganized, newly formed networks remodel coincident with the maturation of regenerated myofibers through 21 dpi [2, 7, 8].

In accord with their spatial proximity, crosstalk between SCs and capillary endothelial cells (ECs) is integral to the regeneration of vascularized skeletal muscle, SC self-renewal, and quiescence [9–11]. Moreover, as the immune response transitions from inflammation and degeneration to a milieu that promotes healing [3, 12], SC proliferation is stimulated [13] and restorative macrophages modulate regeneration by controlling the temporal coupling between angiogenesis and myogenesis [14]. In established models of muscle injury and regeneration [1, 4, 5, 12, 15], basal laminae persist following efferocytosis, which provides cell type-specific guidance to capillaries and myofibers during regeneration [16–21]. Whether microvascular regeneration and myofiber regeneration following injury may proceed sequentially has not been resolved.

In contrast to preserving residual guidance cues following myotoxins or physical trauma, volumetric muscle loss (VML) removes all tissue components. Therefore, as an alternative approach to test the interdependence of microvascular and myofiber regeneration following acute injury, a punch biopsy (diameter, 2 mm) was performed through the mouse gluteus maximus (GM) muscle. This injury is below the critical size threshold of VML that prevents the regeneration of intact muscle [22]. We hypothesized that following a biopsy, angiogenesis would precede myogenesis during regeneration into the wound. The present findings illustrate that microvascular networks develop and become perfused with blood approximately 1 week prior to the regeneration of myofibers.

## Methods

### Animal care and use

Male and female mice (C57BL/6, Jackson Laboratory; Bar Harbor, ME) were bred and housed in animal care facilities of the University of Missouri. Mice (weight, ~ 30 g) were studied when ~ 4 months old. In reporter mice bred on a C57BL/6 background [Cdh5-Cre^ERT2^ [23] x ROSA26^mTmG^ (#007676, Jackson Laboratory)], Cre recombination for expression of membrane-bound green fluorescent protein (GFP) in ECs was induced through intraperitoneal injection of 100 μL of tamoxifen (#T5648, Sigma-Aldrich; St. Louis, MO; 10 mg/mL + 5% ethanol in corn oil) on 3 consecutive days; at least 1 week elapsed after the first injection prior to study. All mice were maintained under a 12:12 h light/dark cycle at 22–24 ºC with fresh food and water ad libitum. To avoid any order effect, the collection of data at criterion timepoints was randomized. Prior to performing a muscle injury, intravital microscopy, or tissue collection, a mouse was anesthetized [ketamine (100 mg/kg) + xylazine (10 mg/kg) in sterile saline; intraperitoneal injection]. Mice were euthanized at the end of an experiment by anesthetic overdose and cervical dislocation.

### Punch biopsy

An anesthetized mouse was positioned on an aluminum warming plate to maintain body temperature at 37 ºC. As needed, supplemental injections of anesthetic (~ 20% of initial) were given to maintain a stable plane of anesthesia as confirmed by lack of withdrawal to a toe pinch (monitored every 15 min). The skin overlying the left GM was shaved and sterilized by swabbing 3 × with betadine and 70% alcohol. While viewing through a stereomicroscope, the mouse was positioned on its abdomen and a ~ 1-cm incision was made through the skin to expose the GM, which was continuously irrigated with sterile saline. A hole was made through the center of the GM with a sterile 2-mm diameter biopsy punch (MediChoice #DP0200, Owens & Minor; Mechanicsville, VA) positioned perpendicular to the muscle surface; remaining adhesions around the circumference of the injury were carefully cut with fine-tipped Vannas scissors to free the biopsy from surrounding tissue. Larger vessels were avoided to minimize bleeding. This volume of muscle loss allows for regeneration [22] while creating a void into which the regeneration of microvessels and myofibers could be studied. Anatomical landmarks provided a reference for consistency in the biopsy site. The skin was closed with sterile 6–0 nylon sutures (UNIFY #S-N618R13, AD Surgical; Sunnyvale, CA). The entire procedure required ~ 20 min. For recovery, the mouse was placed on a heated platform, monitored until consciousness and ambulation were restored (~ 2–3 h), then returned to its original cage. Normal activity and behavior were routinely observed within 24 h. Regeneration of tissue components into the wound was evaluated at designated timepoints through 21 days post-injury (dpi) with uninjured mice (0 dpi) serving as controls.

To evaluate cellular damage at the biopsy site (Fig. S1), Evans Blue dye [EBD [24]; 1% solution in sterile saline; #E2129, Sigma] was injected intraperitoneally (10 μL/g body mass) following the surgical procedure. The GM was dissected (as described below) at 1 dpi, and images of EBD staining were acquired with a 4 × objective on an E800 microscope coupled to a DS-Fi3 camera using Elements software (Nikon; Tokyo, Japan).

### Dissection of the gluteus maximus muscle

A mouse was anesthetized, the surgical area was shaved, the mouse was placed on a warming plate, and the skin overlying the GM was removed with scissors. Exposed tissue was continuously superfused with a bicarbonate-buffered physiological salt solution (bbPSS; pH 7.4, 34–35 °C) containing (in mM) 131.9 NaCl_2_ (Fisher Scientific; Pittsburg, PA), 4.7 KCl (Fisher), 2 CaCl_2_ (Sigma), 1.17 MgSO_4_ (Sigma), and 18 NaHCO_3_ (Sigma) equilibrated with 5% CO_2_/95% N_2_. While viewing through a stereomicroscope, the GM was cut from its origin along the lumbar fascia, sacrum, and iliac crest and then reflected away from the body to view its vascular supply from the ventral surface [7, 25].

### Intravital microscopy

The exposed GM was spread onto a transparent rubber pedestal (Sylgard 184; Dow Corning; Midland, MI) and pinned at its edges to approximate in situ dimensions. Exposed tissue on the mouse was covered with plastic film (Glad Press n’ Seal) to prevent dehydration, and the preparation was transferred to the stage of a Nikon E600FN microscope, where the GM was equilibrated for 30 min while continuously superfused with bbPSS at 3 mL/min. Supplemental doses of anesthetic were given throughout the experimental protocol (duration, 2–3 h) to maintain a stable plane of anesthesia (as above). To assess vascular perfusion, 200 μL of fluorescein isothiocyanate (FITC) conjugated dextran (70 kDa; 10 mg/mL sterile saline) was injected into the systemic circulation via the retroorbital sinus and allowed to circulate for ~ 10 min [7, 26]. The GM was illuminated with a mercury lamp for fluorescence imaging using an appropriate filter cube. Images were acquired through Nikon Plan Fluor 4x/0.13 or Plan Fluor 10x/0.3 objectives coupled to a low light CMOS FP-Lucy camera [Stanford Photonics, Inc.; Palo Alto, CA (SPI)] and displayed on a digital monitor. Time-lapse images were recorded at 40 frames/s using Piper Control software (SPI).

### Confocal imaging of fresh whole-mount muscle preparations

The GM was removed from a Cdh5-mTmG mouse and placed in a custom imaging chamber with the ventral surface facing the objective to optimize the resolution of the microvasculature. A drop of PBS (~ 10 μL) was added to the chamber and the GM was flattened by placing a glass block (2 cm × 2.5 cm × 1 cm; mass, 7.8 g) on the dorsal surface. Images were acquired with a HC PL APO 10/0.40 CS2 objective on an inverted laser scanning confocal microscope (TCS SP8) using LASX software (all from Leica Microsystems; Buffalo Grove, IL). Images were also acquired with a Dragonfly 200 Confocal Microscope System [Andor Zyla camera coupled to a Leica DMi8 microscope with a Leica HC PL APO 10x/0.45 objective using Fusion software (Oxford Instruments; Abingdon, UK)]. To image the entire wound, tile scans (3 × 3 grid encompassing ~ 3.5 mm × 3.5 mm × 200 μm deep) were stitched. Using maximum projection *z*-stacks, 10 random microvessels were chosen within a region of interest (ROI; 493 × 493 μm). Diameters of regenerating microvessels were measured at the midpoint between two branch points and averaged for a given GM; 5–7 GM were analyzed at respective dpi timepoints.

### Immunostaining

An excised GM was immobilized by pinning the edges in the well of a 12-well plate coated with Sylgard 184. After washing with PBS, a region of muscle (~ 5 mm × 5 mm) containing the injury surrounded by undamaged tissue was trimmed and prepared for either whole mount immunostaining or frozen in Tissue Tek OCT compound (VWR International LLC; Radnor, PA) to obtain tissue cross sections for histology.

Whole mount preparations were fixed in 2% paraformaldehyde for 30 min, washed in PBS, and placed in blocking buffer (2% bovine serum albumin, 4% normal donkey serum, 0.5% triton X-100 in PBS) for 30 min. Preparations were incubated overnight at 4 °C with validated primary antibodies: rat monoclonal anti-CD31 [platelet endothelial cell adhesion molecule [PECAM-1; 1:400, clone MEC 13.3, #550,274, BD Pharmingen; San Diego, CA [27]] to identify ECs, rabbit polyclonal anti-CD45 [leukocyte common antigen; 1:200, #ab10558, Abcam; Cambridge, UK [28]] to identify inflammatory cells, and goat polyclonal anti-PDGFRα [1:200, #AF1062, R&D Systems; Minneapolis, MN [29]] to identify fibroadipogenic progenitor cells (FAPs) [30–32]. Preparations were washed in blocking buffer, incubated with secondary antibodies for 30 min, washed again, then rinsed in PBS before transfer to an imaging chamber for confocal image acquisition (as above).

For tissue cross-sections, trimmed GM samples were transferred to a cryomold containing OCT compound with 2–0 black silk suture (length, 2 mm) positioned adjacent to the injury for a visual reference. The cryomold was frozen in isopentane cooled in liquid nitrogen and stored at − 80 °C until sectioning. Cross-sections of frozen GM were cut (thickness, 10 μm) with a cryostat (HM 550 Cryostat, Thermo Scientific; Waltham, MA) at the center of the injury with reference to the silk suture.

For fluorescence imaging, sections were fixed in 4% paraformaldehyde for 10 min and stained with CD31 (1:500, BD Pharmingen), mouse monoclonal anti-myosin heavy chain [1:5, MF-20, Developmental Studies Hybridoma Bank, The University of Iowa Department of Biology; Iowa City, IA [33]], rabbit polyclonal anti-laminin [1:200, #PA1-16,730, Invitrogen; Waltham, MA [34]], and appropriate secondary antibodies (1:400, AlexaFluor, Fisher). Prolong Gold containing DAPI (Fisher) was added before slides were coverslipped. Sections were imaged using appropriate filters on an E800 microscope with a DS-Qi2 camera and Elements Software (Nikon).

The distance devoid of myofibers (i.e., a diameter of remaining visible wound) was measured along GM cross-sections accounting for curvatures within the specimen (Fig. 5A, B). Within this region, the total area of CD31^+^ staining was measured to evaluate microvascular density within the wound that preceded myofiber ingrowth.

### Histochemistry

To visualize collagen deposited within the provisional matrix in whole-mount preparations, the GM was removed, permeabilized in 0.5% Triton X-100 in PBS, and incubated for 1 h with PicroSirius Red [1% Direct Red in saturated picric acid; [35]]. Thereafter, GM was treated for 30 min with 0.5% acetic acid in ddH_2_O and rinsed in 100% EtOH. Images were acquired as described for EBD.

### Statistics

Summary data are displayed for individual GM preparations along with means ± s.e.m. Statistical analyses were performed using Prism 9 software (GraphPad Software, Inc.; La Jolla, CA). One-way ANOVA with Tukey’s post hoc tests was used to determine statistical significance between time points with *P* < 0.05 considered significant. Values for “*n*” refer to the number of GM analyzed; one GM was studied per mouse.

## Results

### Punch biopsy creates a void into which regeneration advances

**Fig. 1:**
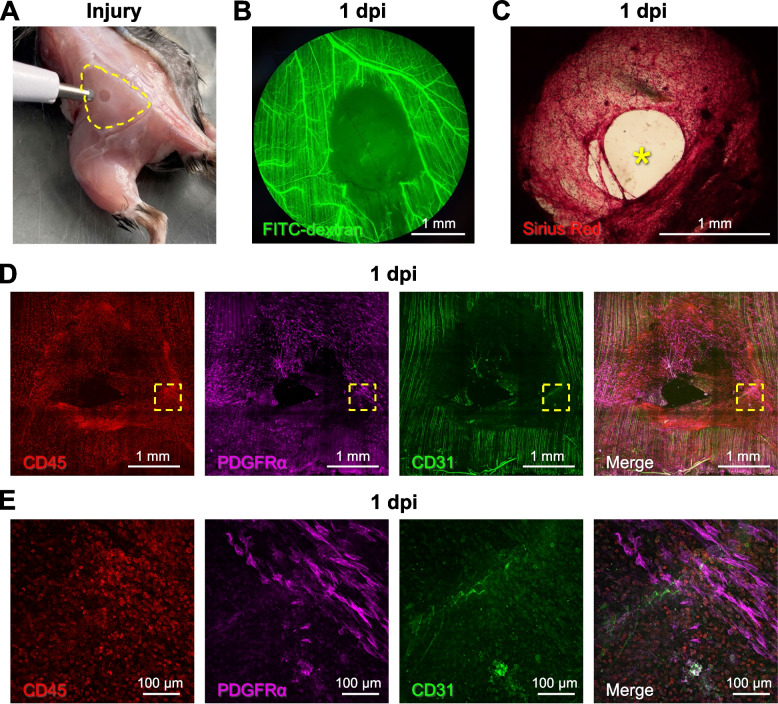
Punch biopsy removes all skeletal muscle tissue components. **A** A biopsy punch was used to create a local injury (diameter, 2 mm) through the GM (muscle perimeter outlined by broken yellow line). **B** Microvascular perfusion was disrupted at the site of the injury at 1 dpi. Intravascular injection of fluorescent dextran showed the empty biopsy site in addition to a small unperfused region downstream of the injury. **C** Representative Sirius red staining of collagen at 1 dpi highlighting deposition of a provisional matrix in the wound; yellow asterisk indicates residual gap. **D** Stitched tile scan of immunostained whole mount GM at 1 dpi showing masses of inflammatory cells (CD45^+^, red) and fibroblasts (PDGFRα^+^, magenta) invading the provisional matrix; capillaries (CD31^+^, green) retain their orientation parallel to uninjured myofibers. **E** Higher magnification at wound edge within broken yellow squares in **D**

Muscle injury was created using a biopsy punch (diameter, 2 mm) to remove a volume of tissue below the critical threshold for regeneration (Fig. 1A; [22]). Creating a circular hole through the center of the GM resulted in minimal collateral damage to the surrounding tissue as evidenced by nominal uptake of EBD into the ends of myofibers bordering the wound at 1 dpi (Supp. Figure 1). Observed in anesthetized mice at 1 dpi, intravascular injection of FITC dextran confirmed the perfusion void within the wound while blood flow was preserved to tissue surrounding the injury (Fig. 1B). Finding that FITC dextran was restricted within the microcirculation and lack of a fibrin clot was consistent with negligible bleeding from microvessels severed by the punch biopsy. Sirius red staining of whole mount GM preparations revealed the deposition of collagen to create a provisional matrix [36] that nearly spanned the wound at 1 dpi (Fig. 1C); immunostaining demonstrated invasion of this matrix by PDGFRα^+^ cells (FAPs) accompanied by CD45^+^ (inflammatory) cells (Fig. 1D), particularly at the edge of the wound (Fig. 1E).

### Microvascular growth into the wound

**Fig. 2:**
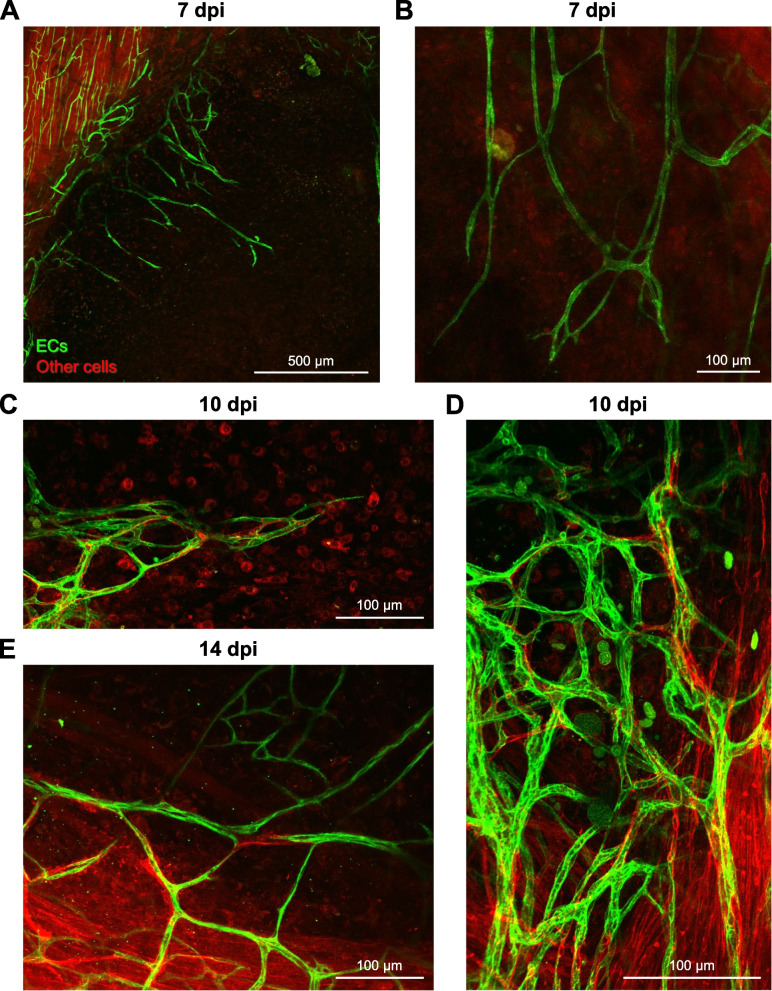
The microcirculation regenerates through angiogenesis. **A** At 7 dpi, nascent microvessels emigrate centripetally from existing vasculature at wound edge. **B** At 7 dpi, microvessels are nonuniform in diameter and extend into the wound with random orientation. **C** Angiogenesis at 10 dpi is integral to revascularization. **D** Sites of robust angiogenic activity at 10 dpi create irregular microvascular plexuses. **E** Regenerating microvascular networks expand at 14 dpi. Images are from Cdh5-mTmG mice in which ECs are green (GFP) and all other cells are red (tdTomato)

Using an EC-specific Cre driver [Cdh5-CreERT2; [23]] and tamoxifen-induced recombination of the Rosa26 mTmG locus to genetically label the endothelium with membrane-bound eGFP (Cdh5-mTmG mice), we evaluated microvascular regeneration following punch biopsy. Endothelial sprouts appeared at 5 dpi (not shown) and extended into the wound at 7 dpi (Fig. 2A, B). From 7 to 14 dpi, the angiogenic response progressed centripetally as nascent microvessels branched into numerous daughter vessels with short interconnections (Fig. 2C–E).

**Fig. 3:**
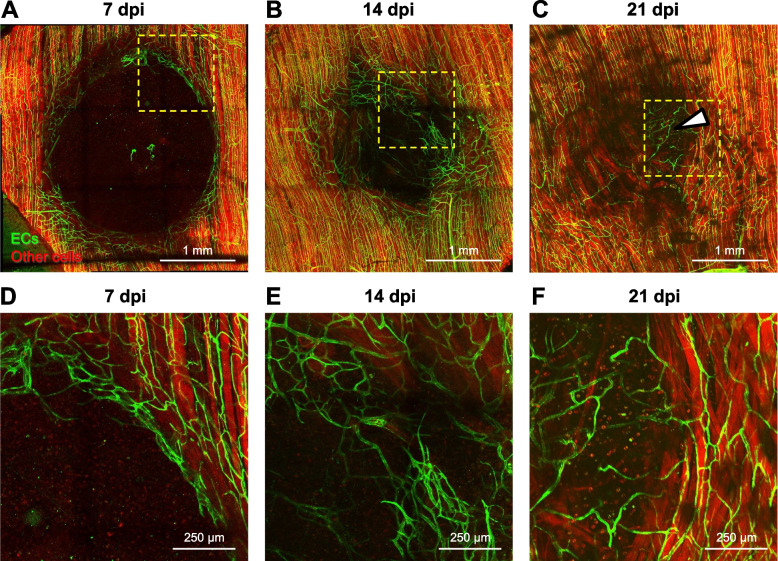
Tile scanned images showing the time course of microvascular and myofiber regeneration after punch biopsy. **A** At 7 dpi, angiogenesis begins around edges of wound. **B** At 14 dpi, regenerating microvessels have spanned the wound and myofibers have begun regenerating into the wound from severed ends. **C** At 21 dpi, myofibers traverse the wound while capillary networks remain disorganized. Dark areas within the regenerated area reflect interweaving among adjacent myofibers, which contrasts with the parallel (flat) organization of surrounding (uninjured) myofibers. Within the wound, microvessels remodel (arrowhead) to supply a cluster of adipocytes (not visible) where myofibers have not regenerated. **D**, **E**, and **F** are higher magnification of regions within broken yellow squares shown in **A**, **B**, and **C** to illustrate angiogenesis at the wound edge, microvascular branches extending across the gap, and microvascular remodeling around a cluster of adipocytes, respectively**.** Images are from Cdh5-mTmG mice in which ECs are green (GFP) and all other cells are red (tdTomato)

**Fig. 4:**
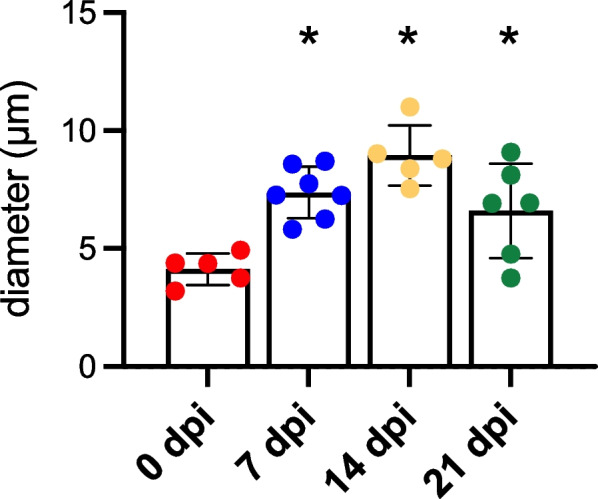
Summary data of microvessel diameters at criterion time points; *n* = 5–7 mice per time point, *P < 0.05 versus 0 dpi

At 14 dpi, microvascular networks spanned the wound and were perfused with blood. However, these newly formed networks lacked hierarchical structure or definitive patterns of blood flow (Fig. 3; Supp. Figure 2). Instead, nascent microvessels were clustered into zones of robust angiogenic activity with random orientation (Fig. 2D). In addition, average microvessel diameter significantly increased from 4.1 ± 0.3 μm in the uninjured muscle to 7.4 ± 0.4 μm at 7 dpi and 9.0 ± 0.6 μm at 14 dpi (Fig. 4). Intravital microscopy revealed that only those microvessels having the largest diameters were continuously perfused; most segments contained stationary red blood cells or plasma alone at 14 dpi (Supp. Figure 2). In perfused microvessels, the direction of blood flow oscillated and was interspersed with periods of flow cessation.

From 14 to 21 dpi, extensive microvascular networks encompassed the site of injury (Fig. 3) with diameters (6.6 ± 0.8 μm) returning towards control at 21 dpi. Nevertheless, the parallel arrangement of microvessels in surrounding the uninjured muscles was not restored. Instead, numerous anastomoses created circuitous pathways of local perfusion.

### Regeneration of myofibers into the wound

In striking contrast to the time course of revascularization, the wound remained devoid of myofibers at 7 dpi (Fig. 3A), a time at which myofiber regeneration would be well underway in other models of skeletal muscle injury [1, 5, 7, 37]. As myofibers began to regenerate into the wound by 14 dpi (Fig. 3B), they were interwoven and coursed along the wound edge before spanning the wound and integrating with undamaged myofibers. Additionally, regenerating myofibers were smaller in width (i.e., cross-sectional area) than in surrounding (uninjured) tissue. At 21 dpi, regenerated myofiber segments intertwined as they spanned the wound (Fig. 3C). In some preparations, clusters of adipocytes occupied space where myofibers had not regrown (Fig. 3C, F, Supp. Figure 3) and capillary networks remodeled in accord with adipocyte morphology (Fig. 3C, F).

**Fig. 5:**
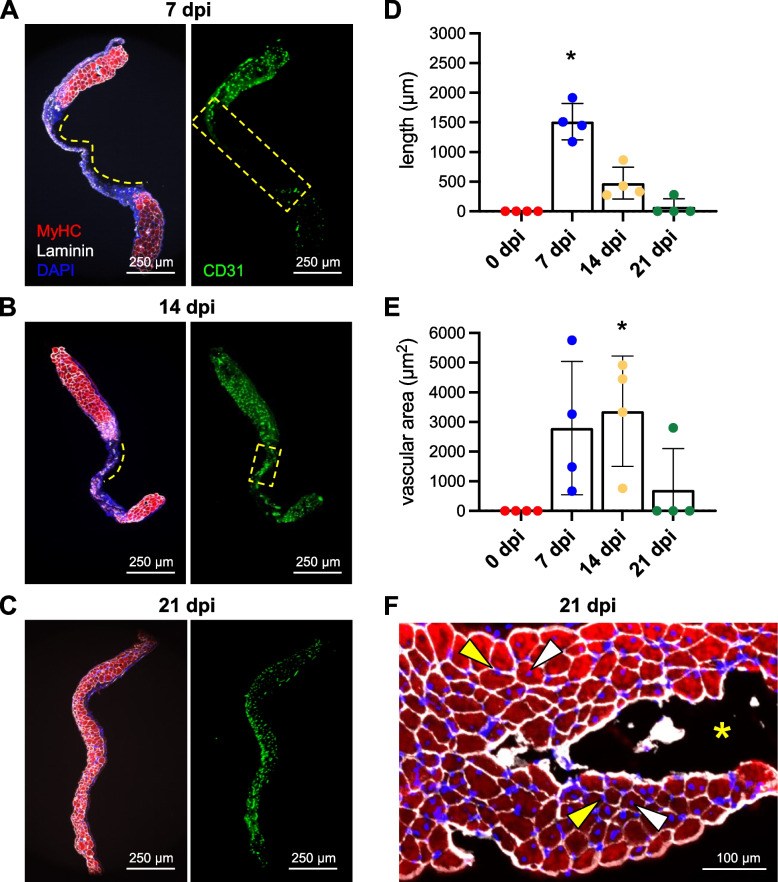
Angiogenesis precedes myogenesis during regeneration. Representative images of GM cross-sections from the center of the wound at **A** 7 dpi, **B** 14 dpi, and **C** 21 dpi. MyHC (myofibers): red; laminin (basal laminae): white; CD31 (ECs): green; DAPI (nuclei): blue. **D** Summary data for length of region devoid of myofibers (shown by broken yellow lines in **A** and **B**). **E** Vascular area within wound region devoid of myofibers (outlined by broken yellow rectangles in **A** and **B**). *n* = 4 per time point, *P < 0.05 vs 0 dpi. **F** Cross-section of from regenerated region within the wound at 21 dpi stained for laminin (white), MyHC (red), and myonuclei (blue). Some myofibers exhibit centrally located nuclei (white arrowheads) while others exhibit peripheral nuclei (yellow arrowheads). Yellow asterisk: region within biopsy where myofibers did not regenerate

To confirm that revascularization occurred prior to myofiber regeneration following the biopsy, GM cross-sections (thickness, 10 μm) were immunolabeled for CD31 to identify ECs, myosin heavy chain (MyHC) for contractile myofibers, laminin to detect basal laminae, and DAPI for nuclei. Congruent with data from intravital and confocal imaging, healing progressed into the wound from 7 dpi through 21 dpi with the regeneration of microvessels preceding that of myofibers (Fig. 5A–C). The length of the injured region devoid of myofibers (corresponding to the diameter of the residual wound) was used as an index of healing over time. This length was 1512 ± 153 μm at 7 dpi which progressively decreased to 475 ± 134 μm at 14 dpi and 71 ± 71 at 21 dpi (Fig. 5D). We quantified the area occupied by ECs within the region lacking myofibers as an indicator of revascularization that preceded myofiber ingrowth. This area was 2794 ± 1126 μm^2^ at 7 dpi, 3366 ± 930 μm^2^ at 14 dpi, and decreased to 700 ± 700 μm^2^ at 21 dpi (Fig. 5E). The decrease from 14 to 21 dpi reflects the growth of myofibers into the wound, which localized with nascent microvessels as regeneration advanced. In contrast to central-located nuclei characteristic of regenerated myofibers [1, 4, 5], adjacent myofibers within the injury often had nuclei positioned at their periphery, adjacent to the sarcolemma (Fig. 5F).

## Discussion

This study used a biopsy punch to create a hole through the center of the mouse gluteus maximus muscle. Key findings are that (1) a provisional matrix infiltrated with CD45^+^ immune cells and PDGFRα^+^ FAPs spanned the void within 1 dpi; (2) nascent microvessels began extending into the matrix from the edges of the wound by 7 dpi, developed into branching microvascular networks that spanned the wound and were perfused with blood by 14 dpi; and (3) myofibers began regenerating into the wound from severed ends by 14 dpi and spanned the wound by 21 dpi. Thus, during skeletal muscle regeneration following a subthreshold VML injury, microvascular growth and perfusion are spatiotemporally dissociated from myogenesis by approximately one week. Nascent myofibers were interwoven and disorganized, as were their associated microvascular networks, indicating loss of local guidance cues during regeneration.

The GM is ~ 200-μm thick at the biopsy site and comprised of mixed fiber type [38], rendering it well-suited for studying microvascular networks in mouse skeletal muscle [7, 8, 25]. Previous studies of the GM following injury with BaCl_2_ show that the microcirculation regenerates and recovers function concomitant with myofibers through 21–35 dpi [7, 8], findings consistent with myotoxin injuries to other muscles of the rodent hindlimb [1, 4, 5, 14]. However, the present findings contrast with studies reporting that capillarization is governed by the size and growth of regenerating myofibers [39] or that upregulation of angiogenic genes occurs during the early stage of muscle regeneration [40]. Furthermore, the spatiotemporal association between SC and EC proliferation and differentiation that is manifesting following myotoxin injury [9, 14] appears to be disrupted following complete removal of the tissue.

### Modulation of SC-EC crosstalk by immune cells and FAPs

Previous studies of muscle injury have shown that regeneration of myofibers and microvessels are concomitant processes mediated by multifaceted crosstalk between SCs, ECs, immune cells, and FAPs in a constantly changing microenvironment [3, 5, 6, 9, 12, 14, 30, 41, 42]. Our finding that CD45^+^ and PDGFRα^+^ cells invade the provisional matrix within 1 dpi following punch biopsy (Fig. 1) is consistent with earlier studies of muscle injury [14, 30, 32, 43]. FAPs reside in the interstitial space adjacent to the microvasculature [31, 32, 44]. Upon injury, FAPs enter the cell cycle to proliferate, localize to the site of tissue damage, and work in concert with ECs to promote myofiber regeneration [6, 32, 43]. Activated FAPs deposit ECM proteins (e.g., collagen), secrete myogenic differentiation factors, and recruit immune cells via paracrine signaling [30, 43]. The ensuing cascade of granulocytes, monocytes, and macrophages produces cytokines and chemokines aimed at clearing cellular debris, promoting self-renewal of SCs, and preventing premature myogenic cell differentiation [3, 41] while creating space for myogenesis to ensue [37]. In such a manner, the microenvironment of the provisional matrix established by FAPs and immune cells serves as the foundation for the regeneration of intact skeletal muscle [36].

### Distinctive features of regeneration following punch biopsy injury

The present data illustrate that, following a punch biopsy, matrix generation and angiogenesis precede myofiber regeneration by at least 1 week. This outcome may be attributed to the complete removal of all tissue components, which contrasts with the persistence of basal laminae following injury from myotoxins or trauma in earlier studies [1, 4, 5, 12, 15, 19, 20]. Indeed, preexisting basal laminae are integral to providing guidance during the regeneration of microvessels and myofibers [16–21]. The ECM aids in cell adhesion, cell-to-cell communication, and differentiation [18, 45]. In addition to providing structure to the ECM, collagen fibers guide the growth and organization of capillaries during regeneration [21]. We suggest that the absence of extracellular structure following muscle biopsy may contribute to disrupting the spatiotemporal correspondence between angiogenesis and myogenesis found here relative to earlier studies [1, 5, 7, 37].

In contrast to the onset of sprouting angiogenesis at 2–3 dpi following exposure to myotoxins [1, 2, 7], endothelial sprouts were not apparent until ~ 1 week following punch biopsy (Fig. 2A). This delay may be attributable to prioritized healing around the edges of the open wound prior to EC migration into the provisional matrix. An even longer delay in angiogenesis (14 dpi) was observed following freezing [1], which kills all cells within the injured region. Nascent microvessels were nonuniformly enlarged through 21 dpi compared to capillaries supplying healthy myofibers in the surrounding tissue (Fig. 2). This finding is inconsistent with data from the GM muscle following BaCl_2_ injury, where vessel diameter returned to baseline values and the structure of capillary networks had nearly recovered at 21 dpi [8]. Enlarged microvessels with irregular blood flow patterns observed within the biopsy wound (Supp. Figure 2) may be explained by the absence of hierarchical organization in newly formed networks as well as the lack established arterio-venous pressure gradients [46], which in turn may reflect the absence of structural and functional cues that may otherwise be provided by the residual ECM. That regenerating myofibers intertwine instead of exhibiting their characteristic parallel alignment also indicates that the provisional matrix lacks the structural basis to reestablish the previous arrangement of myofibers [6, 16, 19] as they regenerate from severed ends.

Whereas centrally located myonuclei have been a hallmark of regenerated myofibers [1, 4, 5], including the GM following BaCl_2_ injury [7], regenerating myofibers that filled the void contained myonuclei positioned centrally in some myofibers while located at the periphery of adjacent myofibers (Fig. 5F). This difference in nuclear localization may be explained by the time course of regeneration, with central nuclei originating during robust myoblast proliferation with myotube growth as compared to later-stage SC self-renewal, when asymmetric division repopulates the SC niche [4] giving rise peripheral myonuclei [47].

### Summary and conclusions

Subthreshold VML following a punch biopsy through the mouse gluteus maximus muscle resulted in microvascular regeneration preceding myofiber regeneration by at least one week. A provisional matrix invested with inflammatory cells and fibroblasts was deposited within the first day post-injury. At 7 dpi, EC sprouts at the edges of the wound oriented centripetally and perfused microvascular networks spanned the wound by 14 dpi. In contrast, myofibers began regenerating into the wound by 14 dpi and filled the wound by 21 dpi. Consistent with other models of injury, myofibers invested with a microvascular supply were restored by 21 dpi. The delayed angiogenic response and even later myogenic response observed here may be explained by the absence of residual guidance cues, which may otherwise promote and coordinate the regeneration of myofibers and their vascular supply [16–21]. Regenerated capillary networks and myofibers within the site of injury were disorganized relative to the uninjured muscle, indicating that the total loss of skeletal muscle ultrastructure also disrupts the morphology of regenerating tissue components.

The findings of this study contrast with injuries in which the basal laminae (and stromal progenitor cells) persist within the injury zone and angiogenesis occurs concomitantly with myogenesis as the inflammatory response resolves [9, 14, 17]. Nevertheless, establishing a vascular supply in the provisional matrix can serve to support the ensuing regeneration of myofibers [5, 6]. The punch biopsy injury thereby provides a novel foundation for investigating the nature and time course of cellular crosstalk among respective tissue components during regeneration of intact skeletal muscle following acute injury in the adult.

## Supplementary Information

**Additional file 1: Supp. Figure 1.** At 1 dpi, Evans Blue dye uptake is restricted to the edges of biopsied myofibers.

**Additional file 2: Supp. Figure 2.** Blood flow within the regenerating wound at 10 dpi was visualized by red blood cell transit during intravital microscopy. A. Monochrome image of regenerating microvessels in Cdh5-mTmG mice. B. Color coding denotes the presence and direction of red blood cell flow.

**Additional file 3: Supp. Figure 3.** At 21 dpi, adipocytes accumulate at the biopsy void if myofibers fail to regenerate.

## Data Availability

The data generated and analyzed during the current study are available from the corresponding author on reasonable request.

## Abbreviations

Cdh5-mTmG: Cdh5-Cre^ERT2^ x Rosa^mTmG^ transgenic mice
dpi: Days post-injury
EBD: Evans Blue dye
ECs: Endothelial cells
ECM: Extracellular matrix
GFP: Green fluorescent protein
FAPs: Fibroadipogenic progenitor cells
FITC: Fluorescein isothiocyanate
GM: Gluteus maximus
PDGFRα: Platelet-derived growth factor receptor alpha
MyHC: Myosin heavy chain
SCs: Satellite cells
VML: Volumetric muscle loss
