# Supplementary figures and images for: Angiogenesis precedes myogenesis during regeneration following biopsy injury of skeletal muscle
Source: Skelet Muscle. 2023 Feb 14;13:3. doi: 10.1186/s13395-023-00313-3 (PMC9926536; doi:10.1186/s13395-023-00313-3)

Supp Fig 1

1 dpi

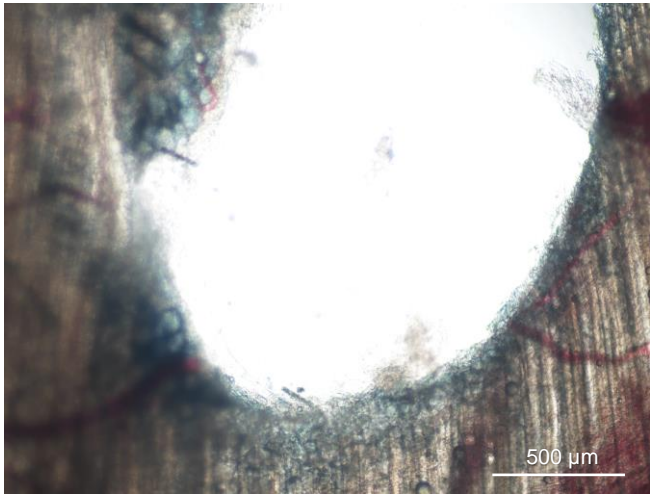

Supplement: Supplementary file 1 — Additional file 1: Supp. Figure 1. At 1 dpi, Evans Blue dye uptake is restricted to the edges of biopsied myofibers. [file 13395_2023_313_MOESM1_ESM.pdf]

## Supp Fig 2

**A**

10 dpi

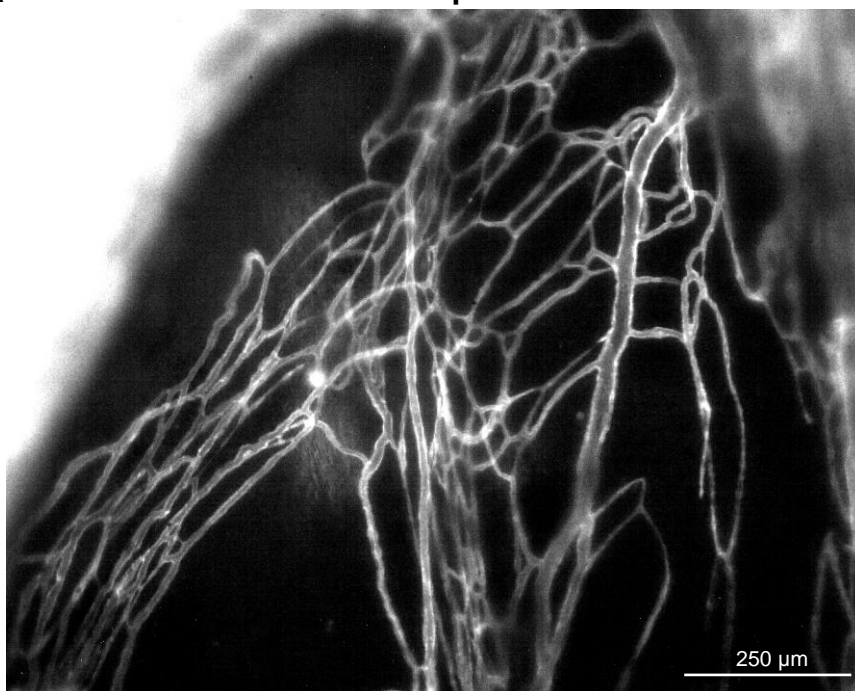

**B**

10 dpi

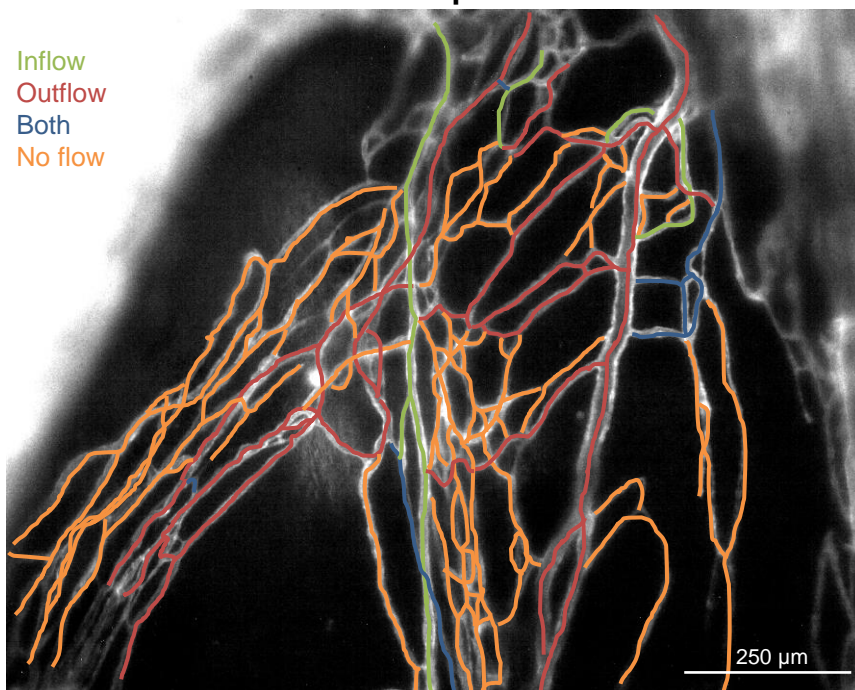

Supplement: Supplementary file 2 — Additional file 2: Supp. Figure 2. Blood flow within the regenerating wound at 10 dpi was visualized by red blood cell transit during intravital microscopy. A. Monochrome image of regenerating microvessels in Cdh5-mTmG mice. B. Color coding denotes the presence and direction of red blood cell flow. [file 13395_2023_313_MOESM2_ESM.pdf]

Supp Fig 3

21 dpi

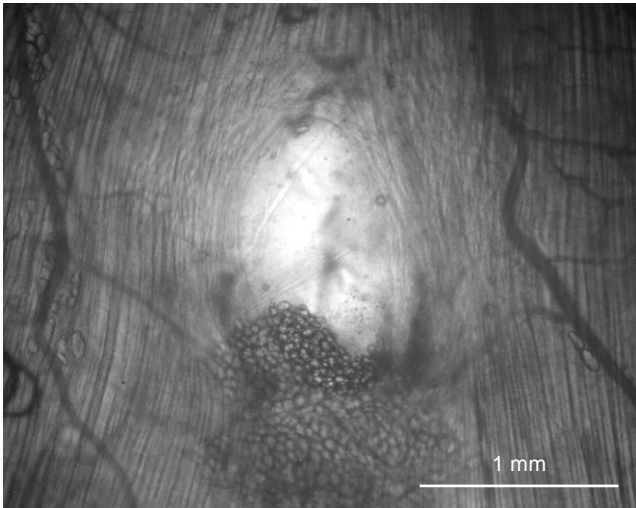

Supplement: Supplementary file 3 — Additional file 3: Supp. Figure 3. At 21 dpi, adipocytes accumulate at the biopsy void if myofibers fail to regenerate. [file 13395_2023_313_MOESM3_ESM.pdf]
